# Nucleic acid-binding specificity of human FUS protein

**DOI:** 10.1093/nar/gkv679

**Published:** 2015-07-06

**Authors:** Xueyin Wang, Jacob C. Schwartz, Thomas R. Cech

**Affiliations:** Howard Hughes Medical Institute, Department of Chemistry and Biochemistry, BioFrontiers Institute, University of Colorado, Boulder CO 80309, USA

## Abstract

FUS, a nuclear RNA-binding protein, plays multiple roles in RNA processing. Five specific FUS-binding RNA sequence/structure motifs have been proposed, but their affinities for FUS have not been directly compared. Here we find that human FUS binds all these sequences with *K*_d_^app^ values spanning a 10-fold range. Furthermore, some RNAs that do not contain any of these motifs bind FUS with similar affinity. FUS binds RNA in a length-dependent manner, consistent with a substantial non-specific component to binding. Finally, investigation of FUS binding to different nucleic acids shows that it binds single-stranded DNA with three-fold lower affinity than ssRNA of the same length and sequence, while binding to double-stranded nucleic acids is weaker. We conclude that FUS has quite general nucleic acid-binding activity, with the various proposed RNA motifs being neither necessary for FUS binding nor sufficient to explain its diverse binding partners.

## INTRODUCTION

FUsed in Sarcoma (FUS, also known as Translocated in LipoSarcoma, TLS), is an abundant nuclear protein that has been implicated in transcription, mRNA splicing and mRNA transport (1–3). Mutations in *FUS* are detected in ∼5% of familial ALS (amyotrophic lateral sclerosis) patients as well as in sporadic ALS (4,5). ALS is a progressive motor neuron disease characterized by loss of the upper and lower motor neurons (6). Patients typically die within 3–5 years after onset of the disease. Dysregulation of RNA is emerging as a pathogenic mechanism in ALS. Therefore, understanding the biology and biochemistry of the FUS protein may provide insights into how this protein can potentially cause the onset of the disease.

FUS, together with EWS (Ewing's sarcoma) and TAF15 (TBP-associated factor 15) in vertebrates, belongs to the FUS/EWS/TAF15 (FET) or TLS/EWS/TAF15 (TET) family (3). The FUS protein has 526 amino acids and is composed of a SYGQ (serine, tyrosine, glycine and glutamine)-rich region at its N-terminus, an RNA-recognition motif (RRM), multiple RGG (arginine, glycine and glycine)-repeat regions, a C2C2 zinc finger motif and a nuclear localization signal (NLS) at its extreme C-terminus. FUS recognition of RNA is mediated by both the RRM and the zinc-finger-containing RGG-Znf-RGG domain (7–9).

RNA binding has been suggested to be crucial for FUS function. FUS inhibits the acetyltransferase activity of CREB-binding protein (CBP) and p300 on the cyclin D1 promoter (10). This inhibition of histone acetylation is dependent on the expression of noncoding RNA *in cis*, and it leads to reduced transcription of the cyclin D1 gene. More generally, our previous work has shown that FUS binds the C-terminal domain (CTD) of RNA polymerase II (RNA Pol II) in an RNA-dependent manner and orchestrates phosphorylation at position Ser2 of the CTD hexapeptide motif (9,11).

Several groups have published RNA sequences that promote FUS binding (9,12–17). One group has utilized *in vitro* SELEX analysis to identify GGUG as a preferred FUS-binding motif (12). However, some RNAs with no GGUG motif are able to bind to FUS (13). More recently, high throughput sequencing has discovered many RNA targets of FUS within the mammalian genome (13–15). Based on these studies, FUS-binding regions of these RNAs have been reported to readily form secondary structures (13–15) and to be enriched either in G/C nucleotides (14,15) or A/U nucleotides (13). However, these reported enrichments represent <10% of the FUS-binding regions. These studies suggest that FUS binding is complicated and that both sequence and structure of RNAs may recruit FUS.

Elucidating the nucleic acid targets of FUS is important for understanding its cellular roles. To characterize the features of RNA targets necessary for FUS binding, we have thoroughly evaluated the binding affinities of FUS with all five published RNA motifs and additional sequences, using electrophoretic mobility shift assays (EMSAs). We found that FUS is able to bind all published RNA sequences within a 10-fold range of binding affinities. In contrast to expectation, however, FUS bound other RNAs including fragments of an *Escherichia coli* mRNA with binding constants similar to those of the published motifs. Consistent with promiscuous binding, we demonstrated that FUS binds RNA in a length-dependent manner. Finally, using competition experiments, we found that FUS had only a modest preference for binding ssRNA relative to single-stranded DNA (ssDNA) of the same length and sequence. We conclude that FUS has a wide range of nucleic-acid binding ability.

## MATERIALS AND METHODS

### Protein expression and purification

The initial FUS expression plasmid was acquired as a gift from the M. G. Rosenfeld lab (UCSD). We added sequences encoding a His_6_-MBP (six histidine-maltose binding protein) tag at the N-terminus of FUS, generating the His_6_-MBP-FUS construct (9). This expression plasmid was transformed into BL21 cells (Life Technologies) and grown in a 5-ml LB-Amp culture overnight. Cultures (1 l) were inoculated and grown at 37°C to OD_600_> 0.8, followed by induction with 0.5 mM isopropyl-beta-D-thiogalactopyronoside (IPTG) and growth for an additional 3–5 h at 37°C. Bacterial cells were pelleted at 6000 rpm for 10 min and lysed in lysis buffer (1 M KCl, 50 mM Tris pH 7.4, 10 mM imidazole, 1 mM CaCl_2_, 5% glycerol, 1% NP40, 1.5 mM β-mercaptoethanol, 1 M urea, micrococcal nuclease (New England Biolabs M02474; 1000 Kunitz Units per gram of cell pellet), followed by sonication (15 s on and 15 s off) for a total time of 1 min. Lysates were cleared by centrifugation at 17 500 *g* for 20 min at 4°C and supernatants were incubated for 1 h with Ni-sepharose beads at 4°C. Beads were pelleted at 2000 rpm for 2 min and washed four times in wash buffer (1 M KCl, 50 mM Tris pH 7.4, 10 mM imidazole, 1.5 mM β-mercaptoethanol, 1 M urea), followed by one time in wash buffer supplemented with 25 mM imidazole. Protein (hereafter called MBP-FUS or simply FUS) was eluted in wash buffer supplemented with 250 mM imidazole. Highly concentrated FUS tends to form aggregates, but the maltose-binding protein (MBP) tag keeps FUS soluble. MBP tag itself does not bind RNA (18). Thus, MBP tags were not cleaved after purification. Our purified MBP-FUS protein was analyzed by size exclusion chromatography, showing high purity and solubility (Supplementary Figure S1A). After purification, the A260/280 ratio was typically in the range 0.57–0.60, indicative of nucleic acid-free protein. The final purified protein (1 mg) was treated with micrococcal nuclease (200 Kunitz Units) and 1.0 mM CaCl_2_ to ensure the complete elimination of nucleic acid and the nuclease was then inactivated by chelating the Ca^2+^ with 1.0 mM ethylene glycol tetraacetic acid (EGTA); we determined that the residual inactivated micrococcal nuclease did not affect the measurement of FUS–RNA binding (data not shown). Protein was aliquoted with 10% glycerol, snap frozen in liquid nitrogen and stored at −80°C.

The percent active protein was determined by titrating MBP-FUS into trace amounts of hot prD RNA and 200 nM cold prDRNA (48 nt) as the substrate. It typically required 1300 nM FUS to fully bind 200 nM RNA. In the case of a 1:1 complex, this would mean that the protein was only 15% active but on the basis of our previous estimate of four FUS molecules per 48 nt RNA (9), the FUS preparation is calculated to be 4 × 15% = 60% active. Here we present *K*_d_^app^ values based on active protein assuming a 1:1 complex so that they are directly comparable to those presented in our previous publication (9), understanding that the real *K*_d_ values are likely to be four-fold higher. Other FUS publications do not report measuring or correcting for the percent active protein.

### *In vitro* transcription of MBP RNA

For MBP 1–10 and MBP 1–20, DNA templates were synthesized by Integrated DNA Technologies (IDT). Complementary strands were annealed and used for *in vitro* transcription. The templates were as follows:

- MBP 1–10 Forward, TAATACGACTCACTATAGGGAGACCAAAACTG
- MBP 1–10 Reverse, CAGTTTTGGTCTCCCTATAGTGAGTCGTATTA
- MBP 1–20 Forward, TAATACGACTCACTATAGGGAGACCAAAACTGAAGAAGGTAA
- MBP 1–20 Reverse, TTACCTTCTTCAGTTTTGGTCTCCCTATAGTGAGTCGTATTA

For other longer MBP RNA constructs, DNA templates were amplified from plasmid pFastBac1 containing the MBP gene from *E. coli*. The primers used were as follows:

- T7-Forward, TAATACGACTCACTATAGGGAGACCAAAACTGAAGAAGGTAAACTGGTAATCTGG
- MBP 1–50 Reverse, CCTTTATCGCCGTTAATCCAGATTAC
- MBP 1–100 Reverse, TTCCGGTATCTTTCTCGAATTTCTTACCG
- MBP 1–200 Reverse, CGGTCGTGTGCCCAGAAGATAATG
- MBP 1–300 Reverse, GTAACGTACGGCATCCCAGGTAAAC

For the MBP RNA bearing the MS2 motif, only MBP1–100 Reverse was changed as follows:

- MBP 1–100 MS2 Reverse: TTCCGGTATACATGGGTAATCCTC

DNA templates for transcription were generated by polymerase chain reaction (PCR) with high-fidelity DNA polymerase (Phusion, NEB). The predicted size of PCR amplicons was confirmed by agarose gel electrophoresis with appropriate DNA size markers. The *in vitro* RNA transcription reactions were set up as described (19). Briefly, the reactions were carried out with T7 RNA polymerase and were incubated at 37°C for 2 h, followed by inactivation at 65°C for 20 min. A trace amount of radioactive CTP [α-^32^P] was included in the reaction to body-label the transcripts. The reactions were spun down and supernatants were treated with RQ1 RNase-free DNase (M6101, Promega) to digest DNA template. The digestions were stopped by addition of 50 mM EDTA. Unincorporated nucleotides were removed by a microspin G25 column (GE Healthcare 27–5325–01). Then, the reactions were mixed with formamide dye, incubated 5 min at 95°C and loaded onto a 10% w/v 29:1 acrylamide:bis 7 M urea gel. The bands containing radiolabeled RNA were excised from the gel and the RNAs were eluted for 1 h at 4°C by 0.3 M sodium acetate, pH 5.2. The eluant was precipitated with glycogen and ethanol at −80°C overnight and the body-labeled RNAs were quantified by liquid scintillation counting.

### End-radiolabeling RNA

prD RNA, GGUG RNA and other RNA oligos were synthesized by IDT and end-radiolabeled with γ-^32^P-ATP using T4 polynucleotide kinase (NEB); incubation was at 37°C for 45 min, followed by inactivation with EDTA. Unincorporated nucleotides were removed and RNA was gel-purified as described for *in vitro* transcription.

### Electrophoretic mobility shift assays

In a 20 μl binding reaction, a trace amount of ^32^P-labeled RNA was incubated with MBP-FUS in binding buffer (50 mM Tris–HCl pH 7.4, 150 mM KCl, 2 mM MgCl_2_, 2 mM Dithiothreitol (DTT), 0.1 mg/ml yeast tRNA, 0.1 mg/ml bovine serum albumin and trace amount of orange dye) at room temperature for 30 min. A portion of each reaction was loaded onto a 4–20% Tris-borate-EDTA (TBE) (Invitrogen EC62252BOX) gel and run at room temperature at 150 V for 70 min. Gels were vacuum dried for 60 min at 80°C and the [^32^P] radioactive signal was detected by exposure to phosphorimager screens. The signals were acquired with a Typhoon Trio phosphorimager (GE Healthcare) and densitometry was quantified with ImageQuant software (GE Healthcare). Quantified data were fit to a sigmoidal binding curve with MATLAB (MathWorks), allowing calculation of both dissociation constants and Hill coefficients.

For competition assays, an appropriate concentration of unlabeled competitor RNA or DNA was mixed with 5000 cpm radiolabeled RNA of the same sequence in a 20 μl reaction. The binding reaction was performed as described above.

## RESULTS

### FUS is able to bind many RNAs

**Figure 1.**
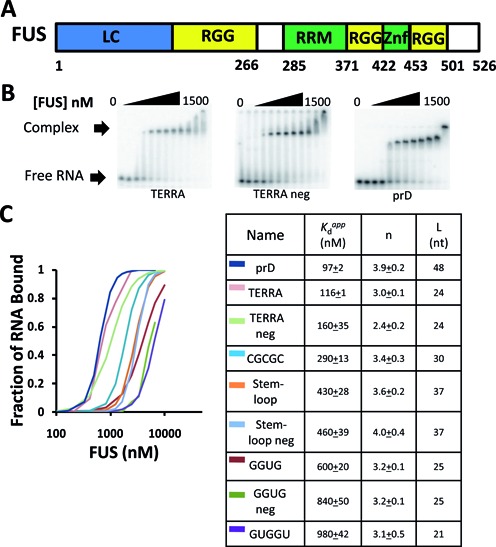
FUS binds many RNAs. (**A**) A schematic representation of the FUS protein. Blue, low complexity domain. Yellow, RGG domains. Green, RNA-binding domains. (**B**) A trace amount of TERRA (left), TERRA neg (middle) or prD (right) was incubated with increasing concentrations of MBP-FUS (0, 15, 31, 62, 125, 188, 250, 375, 500, 750, 1000 and 1500 nM). Binding was analyzed by electrophoretic mobility shift assays (EMSA). (**C**) Summary of RNA binding data for MBP-FUS with nine different RNAs. Left, Quantification of F_bound_ (RNA in complexes per total RNA in lane) as a function of MBP-FUS concentration. Right, the apparent dissociation constant was calculated for each RNA. n and L represent Hill coefficient and length of the RNA, respectively. Uncertainties represent the range of two or more replicates.

Five different RNA sequences have been reported to be preferentially bound by FUS protein (12–17). Among these, GGUG, CGCGC and GUGGU are suggested to contain a specific sequence motif recognized by FUS (12–16). On the other hand, Stem-loop and TERRA form unique secondary and tertiary structures suggested to promote FUS binding (13,17). We hypothesized that FUS may bind one of these RNAs with exceptionally higher affinity than the others. To test this hypothesis, we measured the binding of *E. coli-*expressed FUS protein to eight RNAs including the five published motifs and three negative control sequences (Supplementary Table S1). We also tested prD RNA, one of many human ncRNAs that recruits FUS *in vivo* identified in our previous study (11). EMSA was performed with increasing concentrations of MBP-FUS protein and a trace amount of end-labeled RNA to measure binding affinities (Figure 1A).

Discrete shifted bands were observed, indicating RNA–protein complexes of specific stoichiometry and absence of aggregation. All nine sequences tested were bound by FUS, each with a *K*_d_^app^ in the range between 100 and 1000 nM (Figure 1B and Supplementary Figure S1B). The similar binding affinities of very different RNAs (e.g. CGCGC, stem-loop and GGUG) cast doubt on their specificity for binding to FUS. This skepticism was reinforced by the small differences in affinity between three of the proposed motifs and their mutated forms (cf. TERRA and TERRA neg, stem-loop and stem-loop neg, GGUG and GGUG neg). Furthermore, the prD RNA binds FUS as well as any of the other published RNAs but contains none of the motifs (9).

The EMSA patterns suggested positive cooperativity between FUS and RNA, as it took only two or three protein concentration points to proceed from unshifted RNA to the low-mobility completely shifted complex (Figure 1A). We quantified and fit the binding data with the Hill equation, which revealed that FUS bound each sequence with positive cooperativity (Figure 1B). The low-mobility complex is thought to contain at least four FUS proteins (9) and the fact that intermediates with one, two or three bound proteins do not accumulate is expected for highly cooperative binding. At higher FUS concentrations, the FUS–RNA complexes shifted more toward the well of the gel. This suggests that additional FUS molecules are associated with the RNA in the highly retarded species compared to the initial low-mobility FUS–RNA complex. Alternatively, some of these complexes may contain multiple FUS associated with multiple RNAs.

Our MBP-FUS protein was purified from *E. coli*, while one previous publication carried out EMSA with His_6_-FUS purified from insect cells (13). To test for differences in FUS obtained from these expression systems, His_6_-FUS purified from insect cells was compared with MBP-FUS purified from *E. coli* by EMSA (Supplementary Figure S1C). The two proteins both formed discrete RNA–protein complexes and the protein concentration necessary to shift half of the radioactively labeled RNA was similar. In both cases, the observation of discrete complexes suggests well-folded protein. Therefore, we used MBP-FUS purified from *E. coli* for all remaining experiments.

### FUS binds RNA in a length-dependent manner

**Figure 2.**
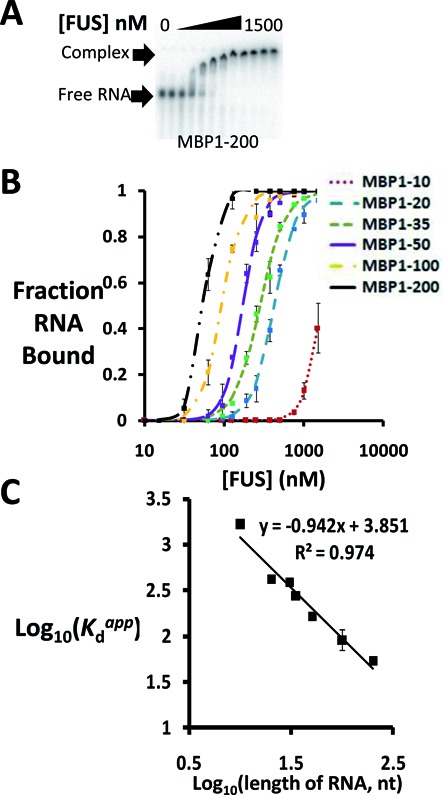
FUS binds RNA in a length-dependent manner. (**A**) EMSA of FUS for RNA containing the first 200 nt of *Escherichia coli* MBP mRNA reveals a tight binding affinity. (**B**) Binding curves were plotted for FUS and RNAs comprising 10, 20, 35, 50, 100 and 200 bases of *E. coli* MBP mRNA. Error bars represent the range of two or three replicates. (**C**) Further analysis of binding curves in (B). Plotting log (*K*_d_^app^) versus log (RNA length) revealed a linear relationship with a slope of −1.

To further test FUS's specificity for RNA binding, we performed EMSAs with portions of the mRNA for the MBP from *E. coli*, an organism that does not possess FUS. Surprisingly, the first 200 nt of the *E. coli* MBP mRNA (MBP 1–200) bound FUS with a reasonably high affinity (*K*_d_^app^ = 56 ± 2 nM; Figure 2A). The electrophoretic mobility of the RNA–protein complex decreased progressively as the FUS concentration was increased, suggesting the loading of more and more FUS onto the mRNA and low binding specificity. The Hill coefficient was 4.8 ± 0.1, indicating that multiple FUS proteins bound this non-human sequence in a positively cooperative manner.

Even though MBP–RNA originates from *E. coli*, it was still possible that some sequence or structure hidden in this RNA could have been responsible for promoting FUS binding. To test this possibility, we *in vitro* transcribed a series of MBP RNAs, including RNA containing the first 10 nt (MBP 1–10), first 20 nt (MBP 1–20) and so on, and then measured their binding to FUS. If MBP 1–200 contained some sequence or structure necessary to bind FUS, then there should be a sudden increase in affinity at the length corresponding to the inclusion of the motif. If no such sequence or structure existed in MBP 1–200, FUS might bind all the truncated sequences.

As shown in Figure 2B, there was no discrete length cut-off for FUS binding, but rather an incremental increase in affinity with increasing RNA length. FUS bound MBP1–20 but not MBP 1–10, defining a minimum length for RNA binding. As the RNA length increased, the binding curves shifted from right to left, indicating an increase in binding affinity (Figure 2B). In other words, *K*_d_^app^ decreased with increasing RNA length. Plotting log (*K*_d_) versus log (RNA length) revealed a linear relationship between dissociation constant and RNA length with a slope of −1 (Figure 2C), consistent with promiscuous binding (20).

### A specific RNA-binding protein is able to recognize its specific RNA motif within a longer RNA

**Figure 3.**
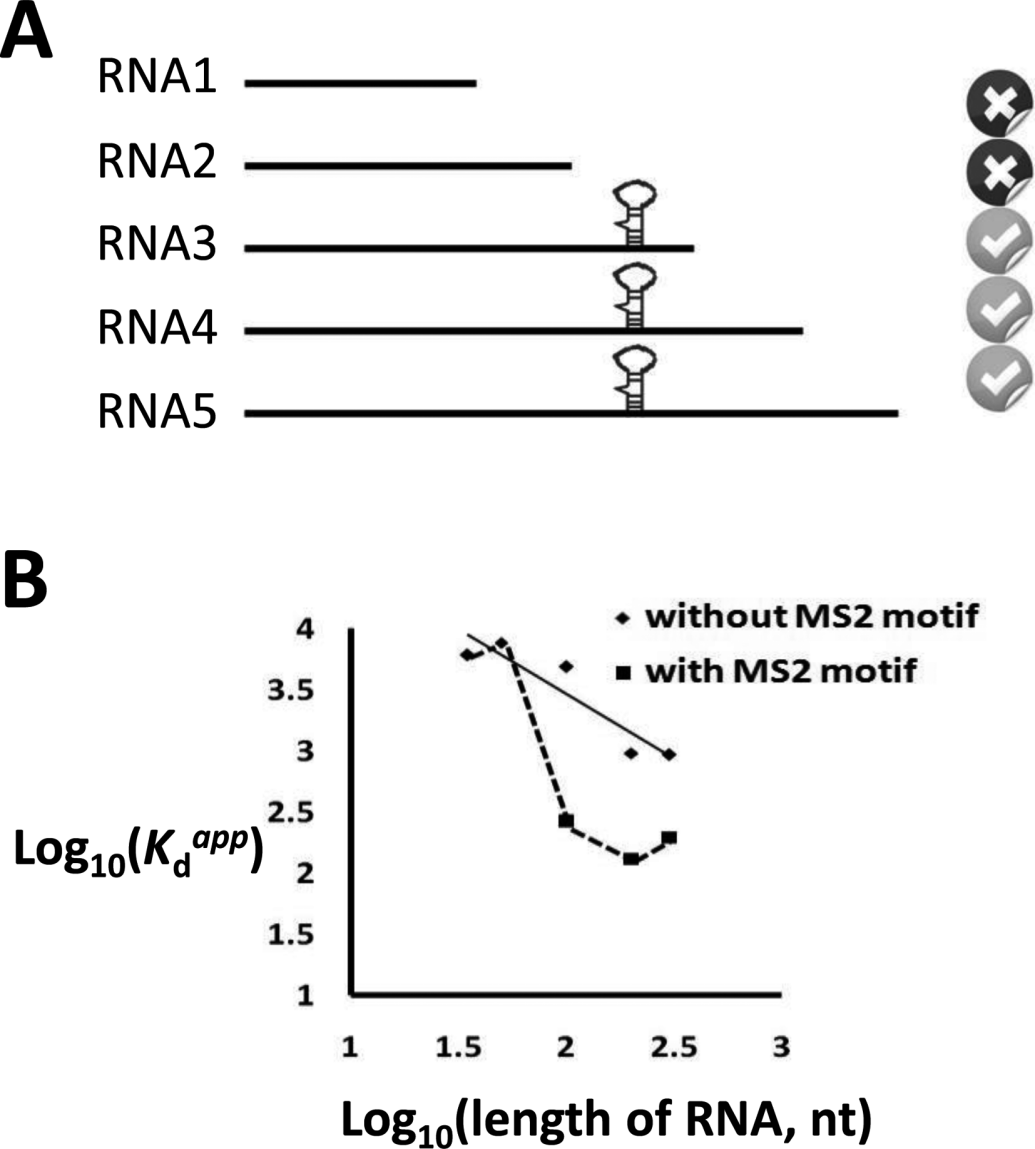
MS2 coat-binding protein is able to recognize its specific MS2 RNA stem-loop motif inserted into MBP 1–200 RNA. (**A**) Schematic representation of the system used. Symbol to the right of each RNA indicates whether motif-specific binding is expected. (**B**) Plotting log (*K*_d_^app^) versus log (RNA length) for RNA sequences with or without the MS2 motif showed more than an order of magnitude drop in *K*_d_^app^. Dash line connecting the second point to the third point shows the difference between the *K*_d_^app^ without and with MS2 motif. Each point represents the average of two or three replicates.

The conclusions above relied on the assumption that a sequence-specific RNA-binding protein can recognize and bind its specific motif hidden in a long sequence and that shorter RNAs without this motif will no longer recruit the protein to bind (Figure 3A). To validate this assumption, we substituted the MS2 recognition motif for a portion of the MBP 1–200 sequence. The MS2 motif forms a stem-loop structure, recruiting specifically MS2 coat protein with a high affinity (*K*_d_^app^ = 4 nM) (21). The position of substituting the MS2 motif was selected from locations where the MS2 RNA can still be properly folded in the context of the MBP long sequence, using the mFold program to predict RNA secondary structures (Supplementary Figure S2).

We *in vitro* transcribed two series of MBP mRNAs, each comprising the first 10 nt, the first 20 nt and so on from the 5′-end of the mRNA. One series had the 21 nt MS2 motif substituted for nucleotides 71–92 and the other did not. RNA sequences in the series lacking the MS2 motif bound MS2 coat protein weakly with a micromolar binding affinity (Figure 3B). In contrast, in the series containing the MS2 motif, the binding affinity increased substantially for the third, fourth and fifth RNAs, which contained the MS2 motif. Even though the binding affinity did not increase all the way down to 10 nM, the *K*_d_^app^ we measured for binding to an isolated 21 nt MS2 motif, the *K*_d_^app^ still dropped dramatically from several micromolar into the 100 nM range (Figure 3B). The reduced affinity of the coat protein for the MS2 site in the context of long RNAs could be due to sampling of multiple RNA conformations, some of which disrupt the motif. Nevertheless, this control experiment supports our conclusion that if there were a specific motif embedded in a long RNA molecule, it could be found by testing the binding of a series of truncated versions of the long RNA.

### FUS binds RNA without requiring a specific sequence or structure

**Figure 4.**
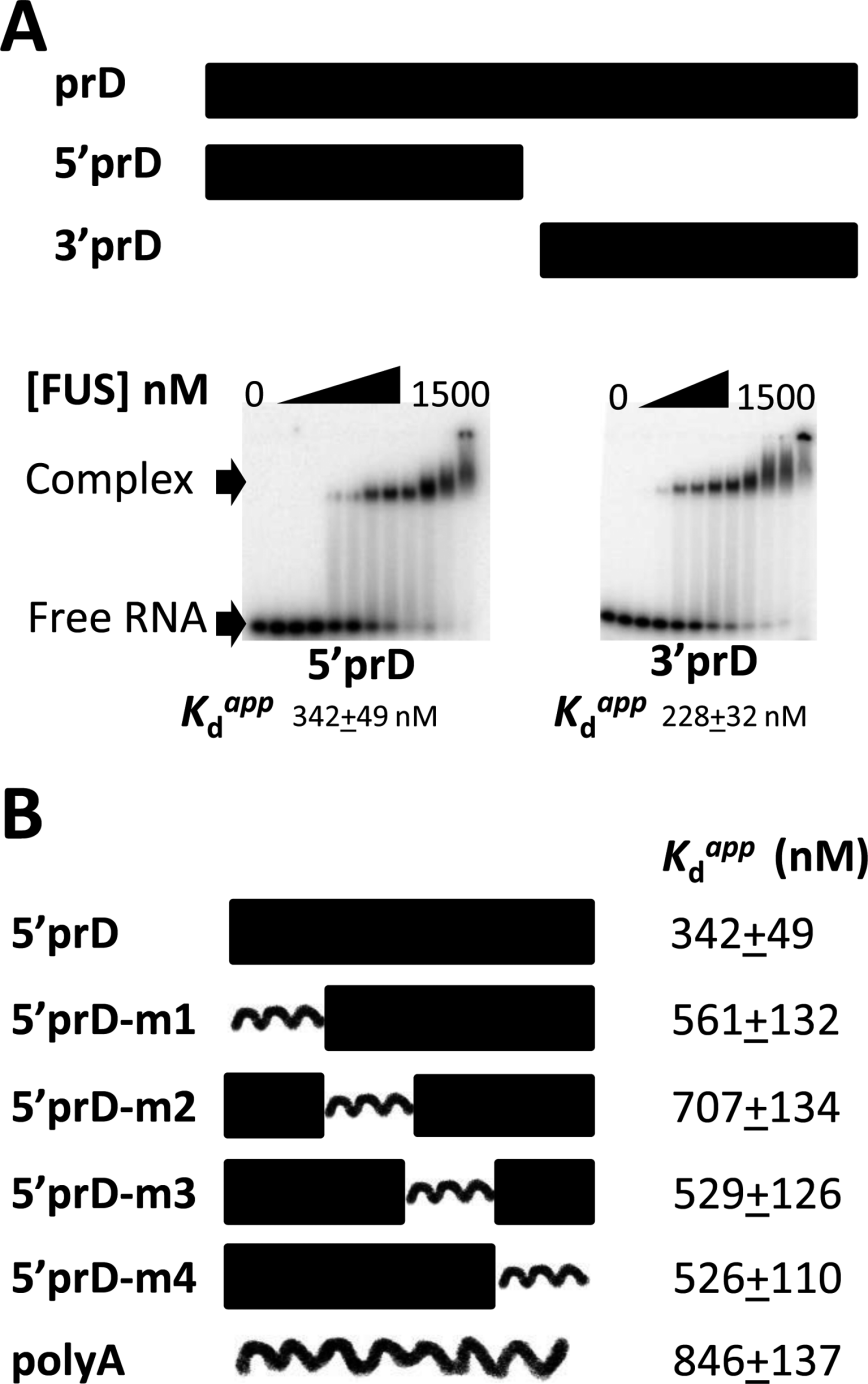
FUS binds the same-length RNA without dramatic difference in binding affinities. (**A**) Top: landscape view of 5′prD and 3′prD (each 24 nt). Bottom: EMSAs between 5′prD or 3′prD and FUS show that both RNA bind FUS equally well. (**B**) FUS binds four mutants of 5′prD as well as the same length polyA. Four mutants of 5′prD were generated by sequentially substituting six nucleotides with six adenines. The binding between each mutant and MBP-FUS was measured and the dissociation constants were calculated based on three replicates. Uncertainty represents standard deviation.

We have observed that FUS is capable of binding many RNAs *in vitro* without dramatic differences in binding affinity and that it binds RNAs such as prD that do not contain any of the published motifs. To further understand the binding of FUS to prD, we divided the 48-mer prD RNA into two 24-mer RNAs named 5′prD and 3′prD (Figure 4A). Both of these RNAs bound FUS with similar affinity and the gel patterns of the two RNAs look identical (Figure 4A), indicating the two RNAs are able to recruit FUS similarly. The reduced affinity for these half molecules relative to prD is expected from the length dependence shown in Figure 2.

Next, we synthesized four additional RNAs by mutating six consecutive nucleotides of 5′prD into a stretch of adenines (Figure 4B). As expected, each of the mutants bound FUS and the *K*_d_^app^ values were in a narrow range between 400 and 800 nM, suggesting that neither sequence nor structure in the 5′prD was required for FUS binding. A polyA 24-mer also bound FUS with an affinity (846 nM) somewhat lower than those of the 5′prD mutants, further indicating FUS does not differentiate dramatically among RNA sequences or structures for binding. In conclusion, FUS binds RNAs with diverse sequences and structures, and when same-length RNAs are compared the binding affinities are within an order of magnitude.

### FUS binds single-stranded DNA and double-stranded nucleic acids with reduced affinity

Previous literature has shown that FUS binds ssDNA and double-stranded DNA (dsDNA) (22,23). To test how well FUS interacts with different forms of nucleic acid, we synthesized six forms of prD each having the same sequence and length (48 nt) as prD RNA. These forms included sense and anti-sense ssRNA, sense ssDNA, dsRNA, dsDNA and DNA/RNA hybrid (Table 1). As expected, FUS bound anti-sense prD RNA. Sense ssDNA bound FUS but with a three-fold weaker *K*_d_^app^ compared to RNA. This is consistent with the observation of weaker affinities with Htelo DNA than TERRA RNA (Supplementary Table S1).Weaker binding to DNA suggests that the 2′ hydroxyl group may contribute to binding FUS. In addition, FUS bound dsDNA and DNA/RNA hybrid more weakly. Surprisingly, full-length FUS was capable of binding dsRNA, indicating FUS is not only a single-stranded nucleic acid binding protein.

**Table 1.** Equlibrium dissociation constants (*K*_d_^app^, nM) for different nucleic acid forms binding to full-length FUS as well as FUS truncations

Name	prD RNA	As-prD RNA	ssDNA	dsRNA	dsDNA	DNA/RNA
FUS	97 ± 2	79 ± 4	280 ± 26	350 ± 38	1200 ± 110	980 ± 100
LC-RRM	60 ± 3	280 ± 22	220 ± 32	2100 ± 60	1400 ± 170	1200 ± 250
RGG-Znf-RGG	81 ± 2	260 ± 32	290 ± 29	1100 ± 80	1000 ± 81	1100 ± 110

Different nucleic acid forms of the prD sequnce were generated and the dissociation constants for binding FUS were measured by EMSA. Each form had the same length as prD RNA. As-prD RNA was the complementary strand of prD RNA and ssDNA had the same sequence as prD RNA. DNA/RNA was a hybrid of ssDNA and As-prD RNA. Two truncations of full length FUS, including LC-RRM (amino acids 1–384) and RGG-Znf-RGG (amino acids 385–526) were made fused with a N-terminal MBP tag. All *K*_d_^app^ values are in nanomolar unit and uncertainties represent the range of two or three replicates.

Previous work demonstrated that both the RRM and zinc fingers of FUS contribute to binding RNAs. We generated two truncations of full-length FUS, including LC-RRM (amino acids 1–384, comprising the low-complexity domain and RNA recognition motif) and RGG-Znf-RGG (amino acids 385–526, containing the zinc finger flanked by arginine-glycine-glycine rich regions). Consistent with our previous work (9), both LC-RRM and RGG-Znf-RGG bound prD RNA with similar affinity as full-length FUS. As expected, both FUS truncations also bound anti-sense prD RNA, although with three-fold lower affinity compared to full-length FUS. Sense ssDNA bound both truncations with similar *K*_d_^app^ as anti-sense prD RNA. In addition, each truncation bound dsDNA and DNA/RNA hybrid very weakly. The FUS truncations, however, bound dsRNA with three- to six-fold higher *K*_d_^app^ compared to full-length FUS, suggesting dsRNA may require both RRM and RGG-Znf-RGG for optimal recognition.

**Figure 5.**
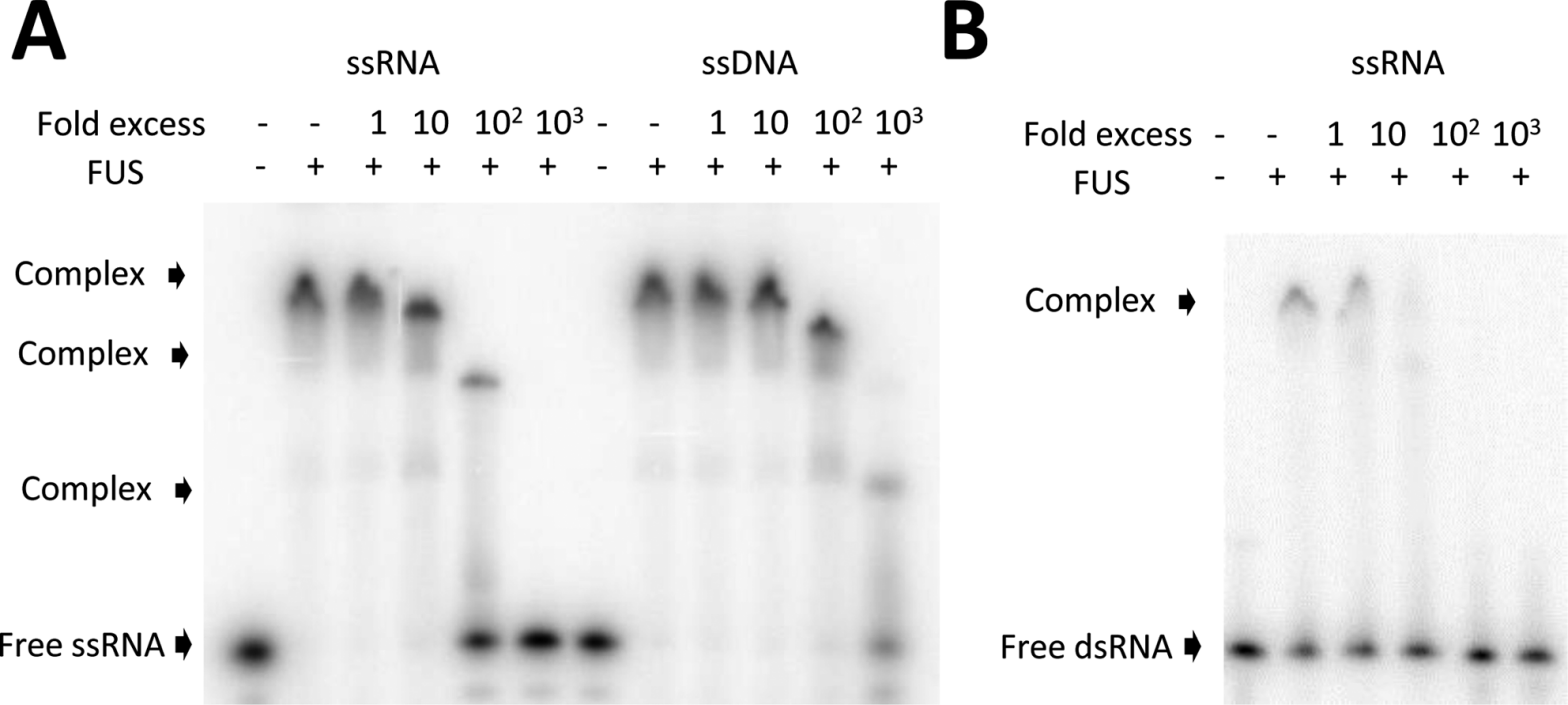
FUS has mutually exclusive binding sites for single-stranded RNA, DNA and dsRNA. (**A**) ssRNA and ssDNA bind FUS mutually exclusively. Increasing amounts of unlabeled ssRNA or ssDNA were mixed with 20 nM radiolabeled ssRNA and the mixtures were incubated with 350 nM FUS. (**B**) ssRNA and dsRNA compete for binding to FUS. The same binding competition assay was performed as in (A) except various amounts of unlabeled ssRNA were mixed with 20 nM radiolabeled dsRNA.

If the same nucleic-acid binding site(s) on FUS bound both ssRNA and ssDNA, then ssDNA should compete for ssRNA binding. To test this, we performed competition assays with radioactively labeled RNA and increasing concentrations of unlabeled ssRNA or ssDNA using EMSA. The radiolabeled prD RNA formed a slow-migrating complex with FUS (Figure 5A). A 100-fold excess of cold ssRNA resulted in displacement of FUS-bound labeled ssRNA (Figure 5A, lane 5). However, a 10 times greater fold excess of cold ssDNA than cold ssRNA was required to displace FUS-bound labeled ssRNA, which is consistent with ssDNA having a weaker binding affinity to FUS. Thus, ssRNA and ssDNA bind to the same site(s) on FUS or they bind to mutually exclusive sites.

Interestingly, new discrete FUS–RNA complexes were observed with addition of cold ssRNA or ssDNA (Figure 5A, lanes 5 and 12), which migrated more rapidly than the original FUS–RNA complexes. This is consistent with multiple FUS proteins being associated with one RNA molecule in the original complexes. The addition of the cold nucleic acid may strip one or two FUS molecules from the original complexes, resulting in the new discrete, fast-migrating complexes. These partially saturated RNAs appear when the competitor nucleic acid concentration is similar to the FUS protein concentration, so there is little if any free FUS protein available to bind the RNA probe. Note that such partially saturated complexes did not appear in the binding experiments with trace amounts of RNA probe (Figures 1 and 4), where FUS is in excess and available for cooperative binding.

We performed the same competition assays between radioactively labeled dsRNA and cold ssRNA. The shifted FUS–dsRNA complexes migrated slightly slower than the FUS–ssRNA complexes. Excess amounts of cold ssRNA competed away the FUS-bound labeled dsRNA (Figure 5B, lane 4), indicating that binding of FUS to dsRNA and ssRNA is mutually exclusive. As dsRNA has a weaker binding affinity than ssRNA, about 10-fold excess of cold ssRNA was required to displace FUS-bound labeled dsRNA compared to the 100-fold excess of cold ssRNA required to displace FUS-bound labeled ssRNA.

## DISCUSSION

The prevailing idea in the FUS field has been that a specific sequence or structure in RNA allows FUS to bind, which led to a simple model by which some motif in RNA recruits FUS functions. Several studies have published RNA motifs (12,13,15–17) that recruit FUS, but their binding has not been tested side-by-side with the same FUS protein preparation. Here, we synthesized all these motifs and measured their *K*_d_^app^ for binding to FUS. We found that FUS is able to bind all of these published motifs, but it also binds to their respective negative controls that disrupt the motifs with only slightly reduced affinity. Furthermore, even *E. coli* MBP mRNA binds FUS and we provide evidence that this is not due to a hidden motif. We conclude that FUS has the ability to bind many RNAs *in vitro* with similar binding affinities and without requiring a well-defined sequence or structure, in agreement with our previous publication (9).

Promiscuous binding of FUS to RNA is consistent with many observations *in vivo*. For example, FUS crosslinks to many thousands of RNAs in cells, including 5′ UTRs, 3′ UTRs and introns (11,13–15,24). Comparison of the RNAs bound to FUS *in vivo* (CLIP-seq) with relative RNA abundance (RNA-seq) shows a definite trend toward FUS binding to abundant RNA (Supplementary Figure S3), consistent with a promiscuous binding component. However, the correlation between FUS-binding and RNA abundance is weak; this is not unexpected, because our biochemical results in no way preclude specific binding of FUS to certain RNAs *in vivo*, e.g. through cooperation with site-specific RNA-binding proteins.

The first published motif (GGUG) was determined based on a SELEX analysis (12). The traditional SELEX technique depended on very low-throughput cloning and DNA sequencing technology and insufficient coverage of all possible sequences or PCR amplification advantages of certain sequences may have contributed to the identification of the motif. Recently the high-throughput RNAcompete method (16) identified the CGCGC motif; we did not find preferential FUS binding to this motif, but this difference could be due to our testing full-length FUS whereas the RNAcompete study tested a truncated FUS (containing the RRM and additional 50 amino acids flanking the N- and C-terminus of the RRM). Two other published motifs were found by transcriptome-wide PAR-CLIP or CLIP-seq analysis (13,15). *In vivo* conditions are different from *in vitro* conditions and FUS binding to the identified motifs *in vivo* may be influenced by other RNA-binding proteins selectively bound to certain RNAs, either precluding or cooperating with FUS binding. Lagier-Tourenne *et al*. (15) conclude that the presence of their GUGGU motif was neither necessary nor sufficient for FUS binding, in agreement with our *in vitro* analysis.

The human telomeric-repeat TERRA RNA (25) can fold into an intramolecular G-quadruplex structure (26). In agreement with the report by Oyoshi *et al*. (17), we found that FUS binds TERRA and in fact it bound with a higher affinity than the other 24-mer RNAs we tested. However, we were unable to confirm the importance of the G-quadruplex structure of TERRA for FUS binding, because in our hands the authors’ mutant ‘TERRA neg’ RNA (containing mutations that would prevent G-quadruplex formation) bound with only slightly reduced affinity (Figure 1). This may perhaps be due to only one FUS concentration being selected for analysis (17) rather than the full binding curves reported here.

We have demonstrated that length of RNA contributes to the binding affinities. However, it is not the sole factor to determine the binding affinities, as we observed two- to seven-fold differences in binding affinities for same-length RNAs. For example, the 24-mers in Figure 4 had *K*_d_^app^ values ranging from 228 to 846 nM and TERRA bound even more tightly with *K*_d_^app^ = 116 nM. This behavior fits the definition of ‘promiscuous binding’: binding to many RNAs without the requirement for an obvious or well-defined protein-binding motif and with affinities that are not enormously different (27).

The Hill coefficients measured here were >1.0, indicating positive cooperatively. Positive cooperativity typically occurs when one protein binding increases the binding affinity for the next protein-binding event via protein–protein interaction. Therefore, multiple FUS proteins appear to be associated with each RNA molecule. This is consistent with our previous conclusion that prD RNA binds at least four FUS molecules (9).

FUS contains two domains that contribute to binding to RNA, the RRM and RGG-Znf-RGG domains (8,9). The RRM in FUS is structurally similar to other RRMs, adopting a canonical β1–α1–β2–β3–α2–β4-fold. However, based on the nuclear magnetic resonance structure of the RRM in FUS, two important aromatic amino acids are missing (7). These two aromatic amino acids normally stack with bases and contribute to specific RNA recognition in the canonical RRM, such as in hnRNPA1 (28). This suggests FUS may interact with RNA in a different way, e.g. through hydrogen bonding with nucleic acid backbones, and lacking these key amino acids may allow FUS to bind many RNAs. We have also demonstrated that both RRM and RGG-Znf-RGG domains bind RNA with similar affinity as full-length FUS. This suggests that FUS may have evolved to have two channels for selecting RNAs, resulting in targeting a much larger variety of RNA.

FUS binds ssDNA. The binding competition results suggest that ssRNA and ssDNA interact with FUS at the same site or overlapping sites. Another possibility is that FUS binding one nucleic acid causes a FUS conformational change that precludes the binding of the other nucleic acid. FUS binding ssDNA is consistent with several observations in the literature. Similar *in vitro* competition results have been shown in other studies (22,29). In fact, FUS protein has been isolated and purified by affinity chromatography on ssDNA (30). The ability of FUS to bind DNA also fits well with its function associated with DNA damage repair. FUS, being one of the earliest proteins recruited to DNA lesions, interacts directly with the DNA repair factors, such as HDAC1 and DNA-PK (31,32). Such interactions are required for successful DNA repair (31,32).

FUS is reported to directly interact with the CTD of RNA polymerase II (11,33). FUS–CTD interactions require RNA, either nascent transcript or noncoding RNA (11). Cooperative binding properties of FUS to RNA may facilitate the formation of higher-order assemblies. These assemblies orchestrate the phosphorylation status of CTD of RNA pol II (9,11,33). Being able to bind many RNAs may allow FUS to target a larger diversity of genes near their transcription start sites, which is consistent with our previous model (11). Also, FUS may bind to long introns and facilitate their splicing (15).

FUS has the intrinsic ability to bind many RNAs without substantial differences in binding affinity, so what determines the FUS interactome *in vivo*? Many other proteins, including hnRNP proteins and splicing factors, are associated with nuclear RNAs and these may preclude FUS binding. On the other hand, some FUS-partner proteins may enhance FUS binding specificity. Similar partner-assisted specificity has been documented for protein–nucleic acid interactions including the homeobox (Hox) family transcription factors, which gain higher DNA sequence specificity and enhanced affinity when paired with an Exd protein partner (34). One can also speculate that post-translational modifications, such as arginine methylation (35), may change the conformation of the protein, conveying preferences for certain RNAs. Future work is required to elucidate the requirements for FUS–RNA interaction in living systems.

## Supplementary Material

SUPPLEMENTARY DATA
