# Supplementary material for: Nucleic acid-binding specificity of human FUS protein
Source: Nucleic Acids Res. 2015 Jul 6;43(15):7535–43. doi: 10.1093/nar/gkv679 (PMC4551922; doi:10.1093/nar/gkv679)
Supplement: SUPPLEMENTARY DATA [file supp_43_15_7535__index.html]

Nucleic acid-binding specificity of human FUS protein — Nucleic acid-binding specificity of human FUS protein — SUPPLEMENTARY DATA 

# Nucleic acid-binding specificity of human FUS protein

## SUPPLEMENTARY DATA

- SUPPLEMENTARY DATA
