# Supplementary material for: Nucleic acid-binding specificity of human FUS protein
Source: Nucleic Acids Res. 2015 Jul 6;43(15):7535–43. doi: 10.1093/nar/gkv679 (PMC4551922; doi:10.1093/nar/gkv679)
Supplement: SUPPLEMENTARY DATA [file supp_gkv679_nar-01151-h-2015-File003.pdf]

Supplementary Table 1

Sequences of RNAs used and the corresponding equilibrium dissociation constants measured by EMSA

Name	Sequence	K_d^{app} (nM)	Structure formed	Putative negative control	K_d^{app} (nM)
**GGUG**	UUGUAUUUUGAGCUAGU UU**GGUG**AC	600±20	N.A.	UUGUAUUUUGAGCUAGU UU**CCUC**AC	840±50
**GUGGU**	CAACUUAGGU**GGUUG**AUUUG A	980±42	N.A.	N.A.	N.A.
**Stem-loop**	GAUUUAUCUUUAACUACUCAA GAUACUGAACAUGACA	430±28	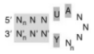	GAUUUAUCUUUAACUACUCU AUCUUCUGAACAUGACA	460±39
**CGCGC**	AGGUCUCAGUUCAU**CGCGC**GA GGUUAUAGU	290±13	N.A.	N.A	N.A.
**prD**	AUUGAGGAGCAGCAGAGAAG UUGGAGUGAAGGCAGAGAGG GGUUAAGG	97±2	N.A.	N.A.	N.A.
**MBP1-200**	See materials and methods section	56±2	N.A.	N.A.	N.A.
**TERRA**	$(UUAGGG)_4$	116±1	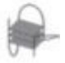	$UUAGGG(UUAGUG)_2UUAGGG$	160±35
**Htelo DNA**	$AGGG(TTAGGG)_3$	730±38	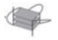	$AGGG(TTAGTG)_2TTAGGG$	690±48

Twelve sequences were used to test whether FUS possesses selectivity to bind nucleic acids. These include seven published sequences, four published negative control sequences and the MBP sequence that originates from *E. coli.* Among these sequences, stem-loop, Htelo and TERRA form unique secondary structures. The binding affinity between FUS and each sequence was measured. The calculated K_d values are listed and uncertainties represent the range of two or more replicates.

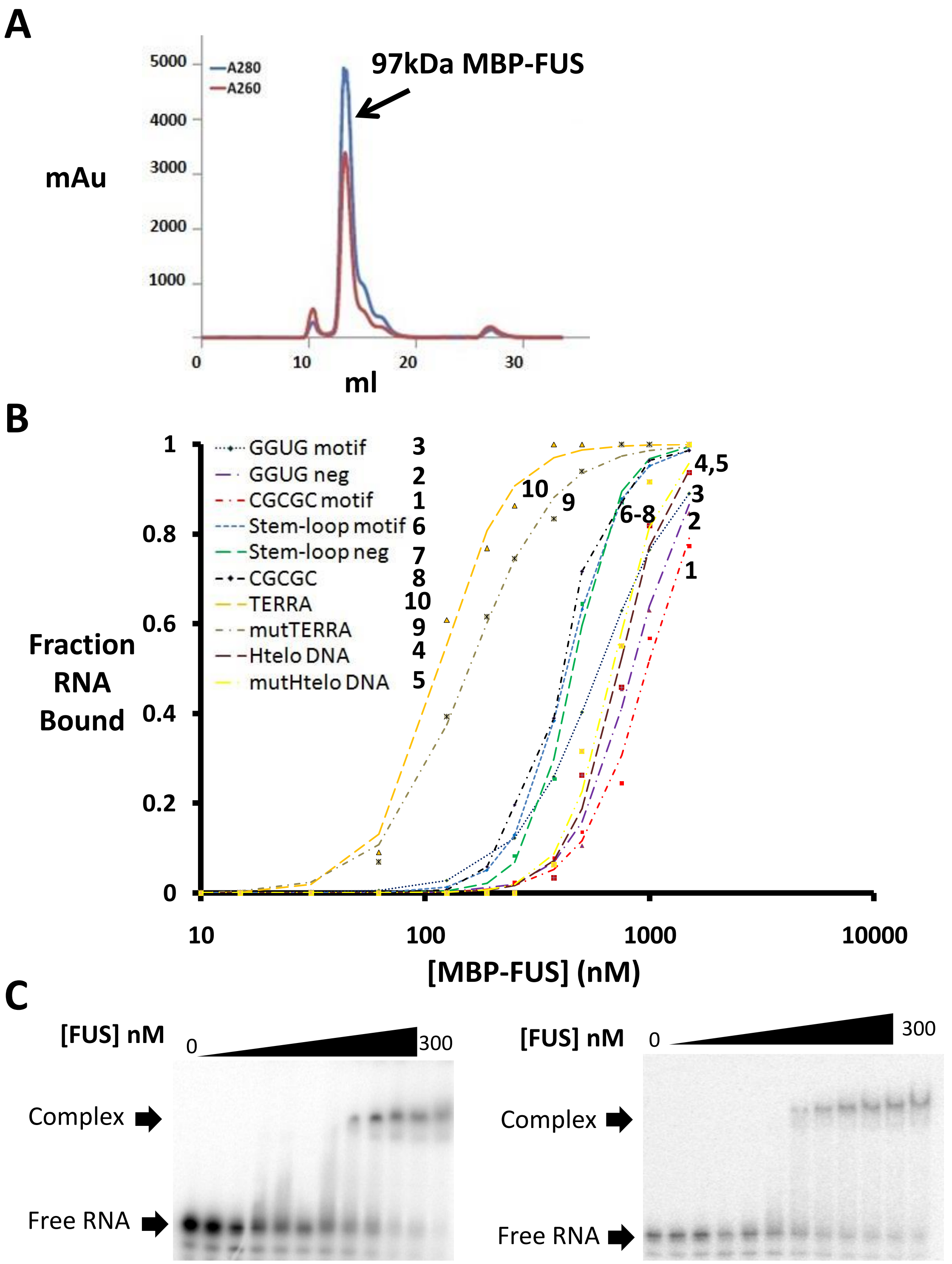

Suppl. Figure 1. (A) MBP-FUS purified by Superdex 200 size-exclusion chromatography has a size corresponding to a monomer, based on comparisons to five standard globular proteins (not shown). (B) RNA binding curves for MBP-FUS with twelve different RNAs. Quantification of F_{bound} (RNA in complexes per total RNA in lane) as a function of MBP-FUS concentration. (C) His_6-FUS purified from insect cells (left) and MBP-FUS purified from *E. coli* (right) were tested for binding to prD RNA. His_6-FUS was expressed in Hi5 cells [11]. The purification was done in the same way as for MBP-FUS.

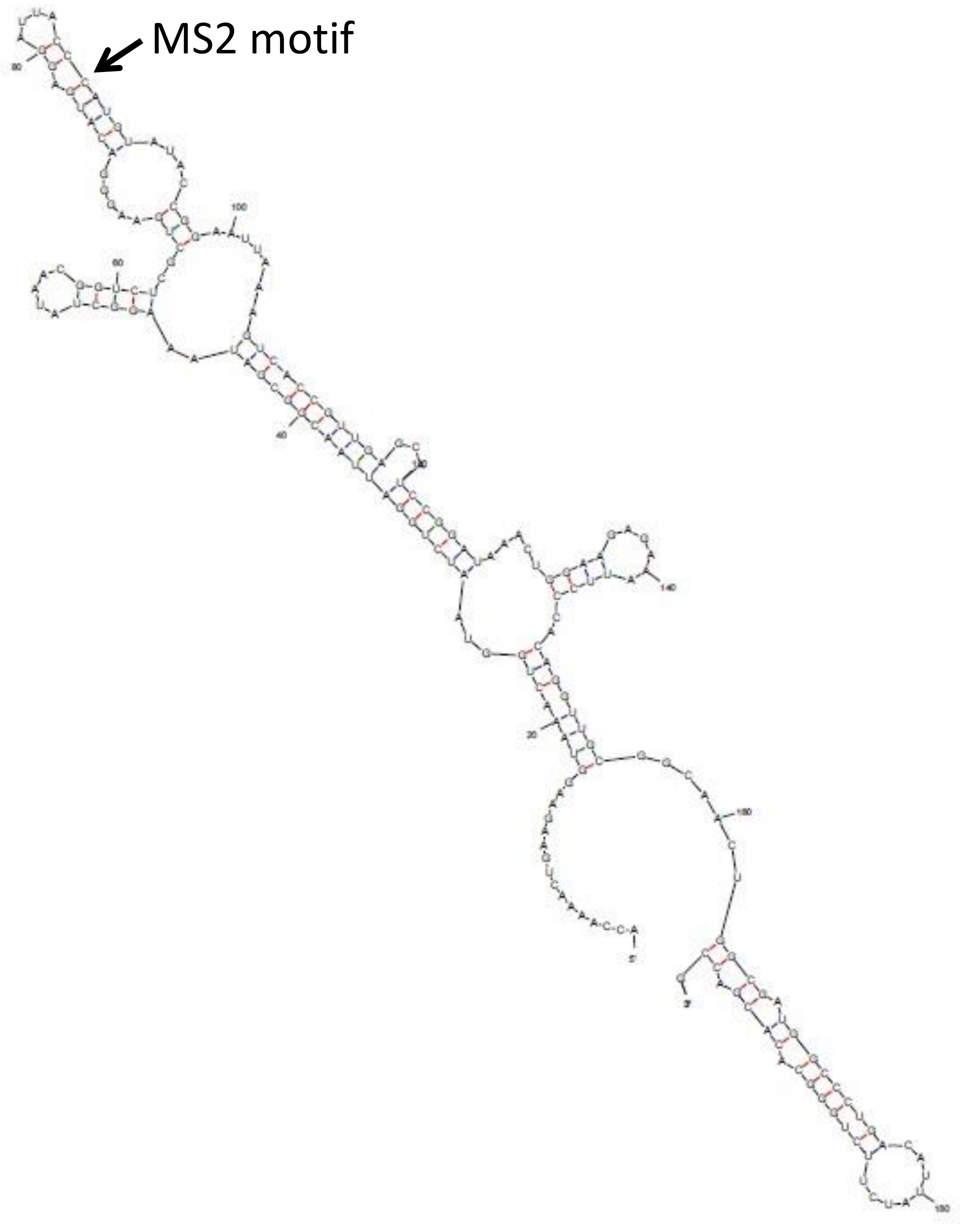

Suppl. Figure 2. The secondary structure of MBP1-200 RNA with an MS2 motif substituted for nucleotides 71-92 as predicted by the mFold program.

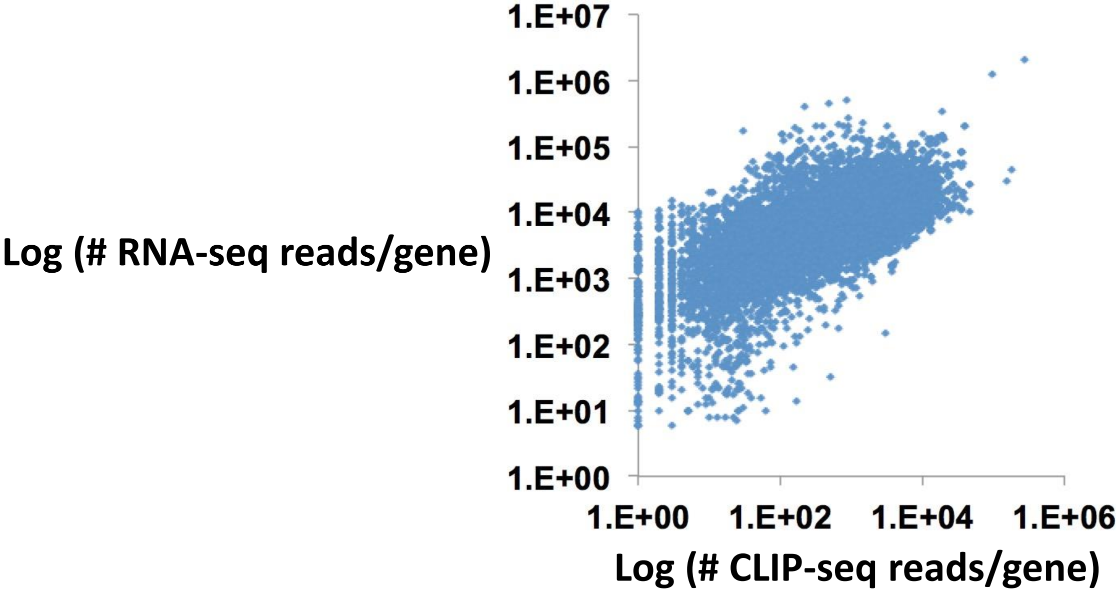

Suppl. Figure 3. Comparing the RNAs bound to FUS in vivo (CLIP-seq) with relative RNA abundance (RNA-seq). Both CLIP-seq and RNA-seq were generated from whole cell extract of HEK 293T/17 cells [11]. Data show a trend towards more abundant RNAs being more frequently bound by FUS (R=0.18). Note that FUS is a nuclear protein, so even in the extreme case that nuclear RNA abundance was the only determinant of FUS binding, that correlation would be imperfectly represented in RNA-seq data on whole-cell RNA.
